# Bilateral osteomyelitis and liver abscess caused by hypervirulent *Klebsiella pneumoniae*- a rare clinical manifestation (case report)

**DOI:** 10.1186/s12879-018-3277-4

**Published:** 2018-08-07

**Authors:** Emma Sturm, Alex Tai, Belinda Lin, Jason Kwong, Eugene Athan, Benjamin P. Howden, Richard D. Angliss, Rafik Asaid, James Pollard

**Affiliations:** 1 Department of Infectious Diseases, Barwon Health, Bellarine Street, Geelong, VIC 3220 Australia; 2 Doherty Applied Microbial Genomics and Microbiological Diagnostic Unit Public Health Laboratory, University of Melbourne at The Peter Doherty Institute for Infection & Immunity, Melbourne, Victoria 3000 Australia; 3 School of Medicine, Deakin University, Geelong, Australia; 4 Department of Orthopaedics, Barwon Health, Bellarine Street, Geelong, VIC 3220 Australia

**Keywords:** *Klebsiella pneumoniae*, Hypervirulent, Osteomyelitis, Pyogenic liver abscess

## Abstract

**Background:**

Hypervirulent strains of *Klebsiella pneumoniae* are a recognized cause of a distinct invasive syndrome that results in pyogenic liver abscesses and metastatic complications, particularly in the Asia Pacific region. Reports of hypervirulent *K.pneumoniae* in Europe, the Americas and Australia indicate worldwide spread. We present a case of multi-focal osteomyelitis, a rarely described complication of hypervirulent *K.pneumoniae* in the medical literature. The prevalence of this condition in countries outside Asia may be expected to rise with increasing travel.

**Case presentation:**

A 20-year-old Chinese man residing in Australia for 2 years presented with a 2-week history of gradually worsening leg pain preceded by 2 weeks of constitutional symptoms. Imaging with computerized axial tomography (CT) and other modalities revealed bilateral tibial lesions described as lattice-like linear lucencies involving the cortices with scalloping of the outer involved cortex. Cultures of tissue from a left tibial bone biopsy were positive cultures for *K.pneumoniae*. Whole-genome sequencing identified the isolate as K1 serotype ST23, a well-recognized hyper virulent strain capable of causing invasive disease. An abdominal CT revealed a 27x22mm liver abscess. The patient had no other metastatic manifestations of the disease, and responded to 6 weeks of intravenous ceftriaxone followed by 3 months of oral Ciprofloxacin.

**Conclusions:**

Increased awareness of the manifestations and subsequent management of hyper virulent strains of *K.pneumoniae* by clinicians is important to assist early recognition and help minimize serious sequelae. Cases with overseas links, such as previous residence in the Asia Pacific area, are at higher risk for infection with the hyper virulent strain. This case highlights the need for clinicians to be able to recognize this important disease, especially in patients with the right epidemiological links, and to investigate and treat appropriately to prevent severe metastatic complications.

## Background

*Klebsiella pneumoniae* is a common pathogen responsible for infections such as urinary tract infections, community acquired pneumonia or surgical site infections. Hyper virulent strains are a distinct population of *K.pneumoniae*, causing the well-recognized “invasive syndrome” that results in pyogenic liver abscesses, as well as extrahepatic metastatic infectious manifestations resulting from bacteraemic dissemination [1]. Rarer complications include necrotizing fasciitis, osteomyelitis, prostatic abscesses and meningitis [2].

Bilateral pyogenic osteomyelitis as a clinical entity is exceedingly rare [3–8] as is long bone osteomyelitis attributable to infection with hyper virulent *K.pneumoniae* [9]. We describe a rare case of bilateral tibial osteomyelitis and liver abscess resulting from infection with hypervirulent *K.pneumoniae* in a previously healthy Chinese student at our Australian health service.

## Case presentation

A previously well twenty-four-year-old student from Hong Kong was referred to our health service with a 2-week history of gradually worsening leg pain and plain film X-ray changes suggestive of an uncharacterized bilateral bony destructive process. The pain was preceded by a 2-week history of constitutional symptoms, including night sweats, chills, fevers, headache, malaise, and a mildly productive cough, which resolved with the onset of bilateral lower limb pain.

There was no past history of Tuberculosis exposure or infection, and routine vaccinations were up to date. The patient reported no overseas travel since his arrival in Australia two years’ prior, denied recreational drug use, and was a non-smoker and drinker. He had no recent animal contacts.

**Fig. 1:**
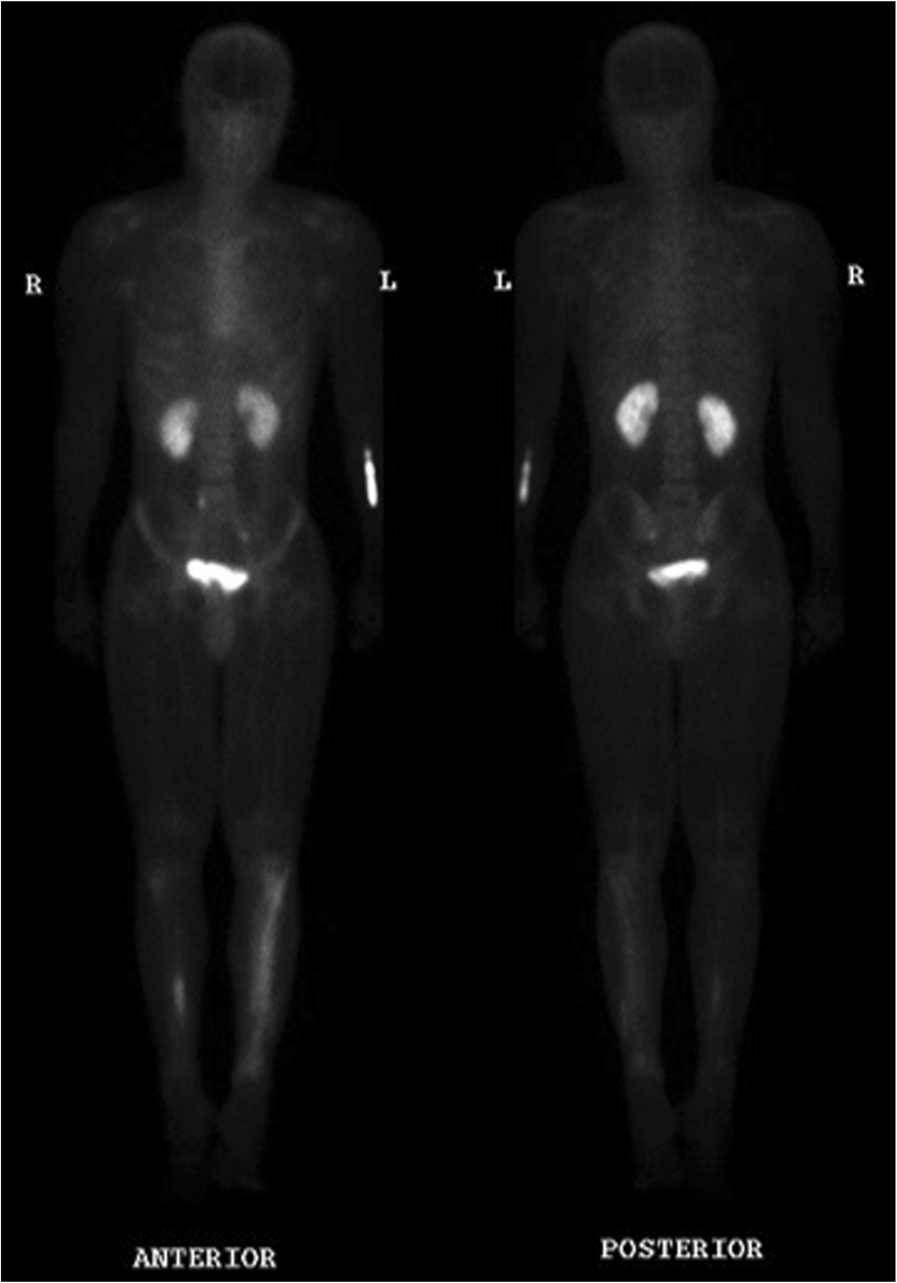
Technetium-99 m bone scan, showing markedly increased tracer uptake of the left tibial and distal fibular shaft, and mid to distal right tibial shaft

Prior to presentation, a course of oral cephalexin was prescribed for presumed cellulitis, which made no difference to his systemic symptoms. On presentation, there was focal pain over the left and right anterior tibial surface. On examination, there was no clinical evidence of a subcutaneous collection. Further imaging of his lower limbs with a computerized axial tomography (CT) scan of both legs revealed bilateral tibial lesions with pronounced changes on the right, described as lattice-like linear lucencies involving the cortices with scalloping of the outer involved cortex. There was no soft tissue mass or periosteal reaction. These changes were further confirmed by a magnetic resonance imaging (MRI) and Technetium-99 m bone scan (Fig. 1). An initially elevated white cell count of 12.2 × 10^9^/L normalized after admission. He had a persistently raised C-reactive protein (CRP), which peaked at 130 mg/L, and an erythrocyte sedimentation rate (ESR) of 88 mm/h. Liver function testing, initially normal, became deranged with a cholestatic picture on day 12 of his admission. A human immunodeficiency virus (HIV) screen and vasculitis screen were negative. Multiple serial blood cultures were negative.

Further investigation with a left tibial bone biopsy was performed, with positive cultures for *K.pneumoniae* on the fresh tissue specimen. The organism was identified using matrix assisted laser desorption ionization-time of flight mass spectrometry ((MALDI-TOF MS, BrukerDaltonics, Biotyper 3.0 database). Antibiotic susceptibility profile was determined using direct colony suspension of 0.5 McFarland on Mueller-Hinton agar. Minimum inhibitory concentration values were interpreted in accordance with Clinical & Laboratory Standards Institute (CLSI) guidelines. The isolate was phenotypically resistant to ampicillin but sensitive to cefazolin, sulphamethoxazole/trimethoprim, gentamicin and ciprofloxacin.

**Fig. 2:**
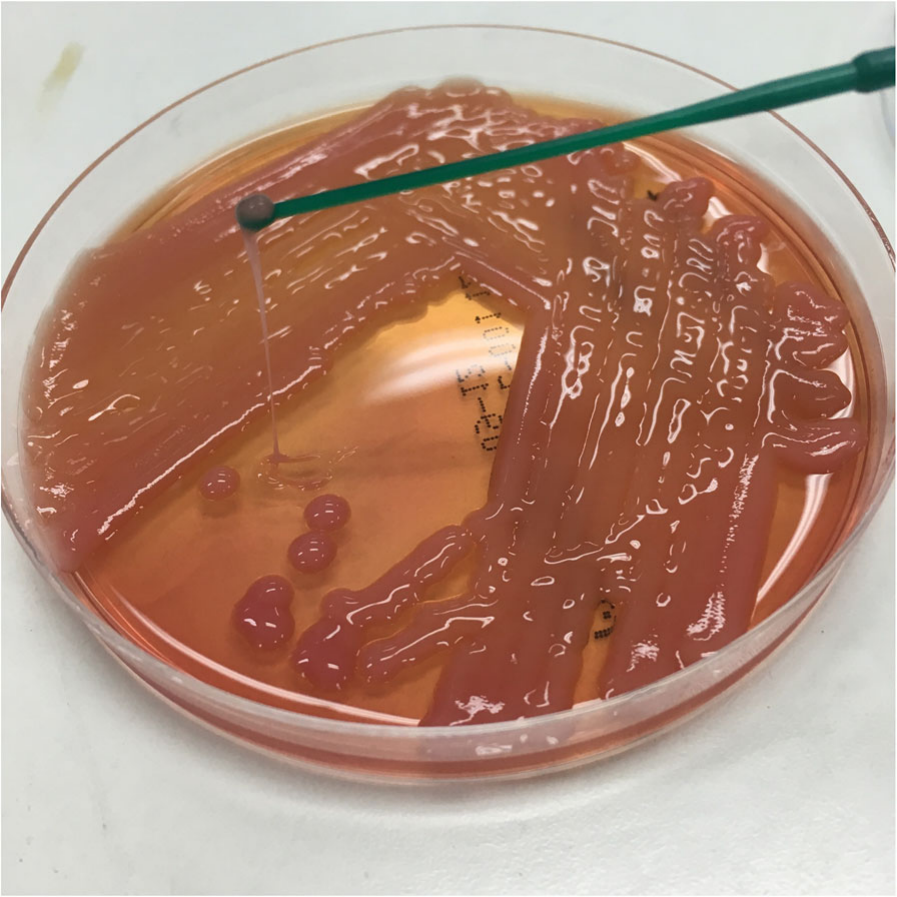
Culture of the isolate from the bone biopsy demonstrating hypermucosity, as evidenced by the formation of a string greater than 5 mm in length using a standard inoculation loop

Whole-genome sequencing of the isolate was performed on the Illumina NextSeq using manufacturer protocols (Illumina Inc., San Diego, CA, USA) [10]. In silico multi-locus sequence typing and analysis of the capsular polysaccharide locus identified the isolate as a K1 serotype, sequence type 23 (ST23) *K. pneumoniae*, a well-recognized hyper virulent strain capable of causing invasive disease. It also possessed a hypermucoid phenotype demonstrated by string test positivity (Fig. 2). Several other virulence genes and operons were also detected, including the mucoviscosity genes *magA*, *rmpA* and *rmpA2*, the allantoin anaerobic assimilation operon *allABCDRS*, the ferric uptake system *kfuABC*, and the yersiniabactin, aerobactin (including the *iutA gene*), siderobactin and colibactin siderophore systems. The only antibiotic resistance elements detected were *bla*_SHV-36_ and *fosA*.

Subsequent CT of the abdomen performed to investigate for a gastrointestinal source of his infection revealed a 27x22mm lesion in segment 8 of the liver, consistent with liver abscess and confirmed on ultrasound, which was not amenable to surgical drainage. On inpatient Ophthalmology review the patient had no signs of endophthalmitis. He was commenced on a 6-week course of intravenous (IV) ceftriaxone at 2 g daily.

Subsequently, his pain markedly improved. Treatment was well tolerated and after 6 weeks of therapy inflammatory markers had normalised, with only minor persistent pain and erythema in the left leg. He then completed a 3-month course of oral Ciprofloxacin with no further evidence of disease relapse. After 3 months the patient was lost to follow up, precluding repeat imaging to assess for resolution of his liver abscess.

## Discussion

The first described case series of *K.pneumoniae* pyogenic liver abscess with metastatic characteristics in the form of endophthalmitis was from Taiwan in the 1980s [11]. The organism has since been recognized as causing a severe metastatic syndrome in previously healthy hosts, particularly in the Asian Pacific region. The organism is now the main cause of liver abscesses in Singapore, Hong Kong, South Korea and Taiwan [12]. Increasing global travel has resulted in reported cases from Europe, the Americas [9] and Australia [13, 14], indicating worldwide spread of the disease.

Hypervirulent strains of *K.pneumoniae* have traditionally been susceptible to most antibiotics compared with the carbapenem resistant strains that have caused large outbreaks in the nosocomial setting [15–17]. However, a variety of resistance mechanisms, including proposed transmission of mobile genetic elements between “classic” and hypervirulent strains of *K.pneumoniae* [18], have resulted in outbreaks of carbapenem-resistant hypervirulent strains in China [18, 19] and other parts of the world [18]. These strains pose a substantial threat to human health because they have potential to cause invasive and severe disease, are highly transmissible and are extremely difficult to treat.

A combination of specific microbiological and host attributes may contribute to disease development. Proposed diagnostic clinical criteria for the invasive syndrome of *K.pneumoniae* include *probable* invasive nature, with isolated liver abscess, or *definite* invasive, which comprises extra-hepatic manifestations [12]. Microbiological definitions of *probable* and *definite* invasive disease include string test positivity, and identification of K1 or K2 serotypes, respectively.

The isolate from this case was of capsular serotype K1, a sequence type 23 strain, with confirmed presence of the wzy-K1 gene (previously magA), now known to encode serotype K1 capsule formation [20]. ST23 is the strain most frequently isolated from invasive infections [21]. The emergence of virulence in these isolates of the same lineage have occurred with the presence of siderophores, demonstrating that the acquisition of iron scavenging systems are crucial in the ability of *K.pneumoniae* to cause invasive disease [21]. The presence of the ‘mucoid regulator genes’, *rmp*A and *rmp*A2, which can upregulate capsule production has also been associated with hypervirulence and, phenotypically, string test positivity [22].

The Chinese ethnicity of our patient has also been demonstrated to be a risk factor for invasive disease [23]. Studies have linked carriage of virulent strains of *K. pneumoniae* in the gastrointestinal tract of healthy hosts as a predisposing factor for development of pyogenic liver abscesses in patients of Asian descent [24, 25]. Although faecal analysis was not completed on our patient, this may have been a predisposing factor in our patient.

A combination of these genes in the ST23 strains and factors related to ethnicity have likely resulted in the emergence of the hypervirulent phenotype in our case, with evidence of pyogenic liver abscess and multi-focal osteomyelitis.

Though well described in children, acute osteomyelitis caused by haematogenous spread is uncommon in adults with no underlying co-morbidities [26, 27]. Acute bilateral long bone osteomyelitis from a pyogenic cause is rarely encountered in clinical practice. Although an established complication of invasive disease, there are limited published cases of primary osteomyelitis caused by invasive *K.pneumoniae* [9]. The possibility of multifocal osteomyelitis was initially raised in this case based on the clinical history of preceding constitutional symptoms and the imaging findings. A malignant process such as osteosarcoma was deemed less likely given the absence of aggressive periosteal reaction or expansive medullary cavity mass on imaging. Given its atypical presentation, this case presented an initial diagnostic challenge requiring careful clinical and radiographic correlation, as well as consideration of the relevant epidemiological background.

## Conclusions

In an increasingly globalised world, our study highlights the importance of clinician awareness of the manifestations and management of *K.pneumoniae* hypervirulent strains, even in areas with few described cases. Further testing and prompt evaluation for the hyper virulent strain in the right clinical context is important to reduce the morbidity and mortality. This is even more crucial with the emergence of carbapenem resistant strains of hyper virulent *K.pneumoniae*. Multi-focal osteomyelitis caused by *K. pneumoniae* is rarely described in the literature. Our case contributes to the growing awareness of the unusual manifestations of this serious disease.

## Abbreviations

CLSI: Clinical & Laboratory Standards Institute
CRP: C-reactive protein
CT: Computerized Axial Tomography
ESR: Erythrocyte Sedimentation Rate
HIV: Human Immunodeficiency Virus
IV: Intravenous
*K. pneumoniae*: *Klebsiella pneumoniae*
MRI: Magnetic Resonance Imaging
ST23: Sequence Type 23
